# Informed Decision-Making with and for People with Dementia—Efficacy of the PRODECIDE Education Program for Legal Representatives: A Randomized Controlled Trial (PRODECIDE-RCT) and Process Evaluation

**DOI:** 10.3390/geriatrics9030060

**Published:** 2024-05-09

**Authors:** Julia Lühnen, Burkhard Haastert, Tanja Richter

**Affiliations:** 1 Unit of Health Sciences and Education, Faculty of Mathematics, Computer Science and Natural Sciences, Universität Hamburg, Martin-Luther-King-Platz 6, 20146 Hamburg, Germany; tanja.richter@uni-hamburg.de; 2 Charité—Universitätsmedizin Berlin, Corporate Member of Freie Universität Berlin and Humboldt Universität zu Berlin, Institute of Clinical Nursing Science, Charitéplatz 1, 10117 Berlin, Germany; 3 mediStatistica, Zur Waldesruh 25, 42329 Wuppertal, Germany; haastert@medistatistica.de

**Keywords:** proxy decision-making, dementia, legal representatives, education program, informed decision, evidence-based medicine

## Abstract

Legal representatives take a major role in healthcare decisions with and for people with dementia, but only a minority has a qualification in this field. The aim was to evaluate the efficacy of the PRODECIDE education program for legal representatives. In a prospective randomized controlled trial, legal representatives (volunteers and professionals, representing at least one person with dementia) were allocated (1:1 computer-generated block randomization) to the intervention (PRODECIDE education program) and control (standard care) groups. The primary outcome measure was knowledge, operationalized as the understanding of decision-making processes and in setting realistic expectations. Only data entry and analyses were blinded. A process evaluation in a mixed methods design was performed. We enrolled 218 legal representatives, and 216 were included in the primary analysis (intervention *n* = 109, control *n* = 107). The percentage of correct answers in the knowledge test post intervention was 69.0% in the intervention and 43.4% in the control group (difference 25.6%; CI 95%, 21.3 to 29.8; *p* < 0.001). In the comparison of professional and voluntary representatives, professionals had 13.6% (CI 95%, 8.0 to 19.2; *p* < 0.001) more correct answers. The PRODECIDE education program can improve the knowledge of legal representatives, an important prerequisite for evidence-based, informed decision-making.

## 1. Introduction

Approximately 1.3 million people in Germany have been appointed a legal representative [1]. The German guardianship system provides a supportive instrument for the legal protection of adults, without limited maturity or restriction of legal capacity [2]. A legal representative can be appointed by court for certain groups of tasks, such as healthcare affairs, if an adult is no longer able to handle his or her own affairs [2,3]. This task requires them to take a major role in decision-making processes [3]. Only a minority of legal representatives have a qualification in the field of healthcare [4]. Therefore, core competencies to support healthcare decision-making cannot be presupposed.

Behavioral and psychological symptoms are common in dementia [5,6,7] and may lead to distress in both persons with dementia and carers [8]. Interventions such as artificial nutrition via a percutaneous endoscopic gastrostomy (PEG), the use of physical restraints (PRs) and antipsychotic drugs (APs) are frequently applied because of anticipated benefits (e.g., prolonged survival due to better nutrition, less falls and injuries and decreased agitation or aggressive behavior) [9,10,11,12,13]. However, evidence for the claimed benefits is weak or controversial, and all interventions have a substantial potential for harm [9,10,11,12,14]. We therefore assume that the application is often not appropriate. Guidelines do not recommend the use of these interventions or only in special situations and with a short duration [15,16,17].

There are strong indicators that these autonomy-restricting interventions are directed more towards facilitating nursing care rather than towards enhancing the quality of life of people with dementia [18]. Physicians are asked—but they should avoid—providing healthcare treatments contrary to good clinical practices and against clinical guidelines. In addition to the question of lack of efficacy, ethical considerations, also regarding the decision-making process, must be considered, both by physicians and by representatives such as legal guardians [19].

We developed the PRODECIDE education program for legal representatives to enhance their competencies in informed decision-making processes and tested it for feasibility [20]. 

The main objective of this study was to evaluate the efficacy of the PRODECIDE education program. The key hypothesis was that, compared to those allocated to the control group, legal representatives allocated to the education group would achieve a better understanding of decision-making processes and higher levels of realistic expectations regarding the probabilities of benefits and harms of a PEG, PRs and APs in people with dementia. These are prerequisites for informed and evidence-based decisions. Therefore, a further objective was to determine whether the education program could result in a reduction in PEGs, PRs and APs in persons with dementia. 

We performed a process evaluation to describe the implementation of the intervention and to understand barriers and facilitators. To support implementation further, we developed a concept for translating the educational contents into e-learning modules. 

## 2. Materials and Methods

The reporting of this study follows the Consolidated Standards of Reporting Trials (CONSORT) Statement for Randomized Trials of Nonpharmacologic Treatments [21]. 

### 2.1. Design

**Figure 1:**
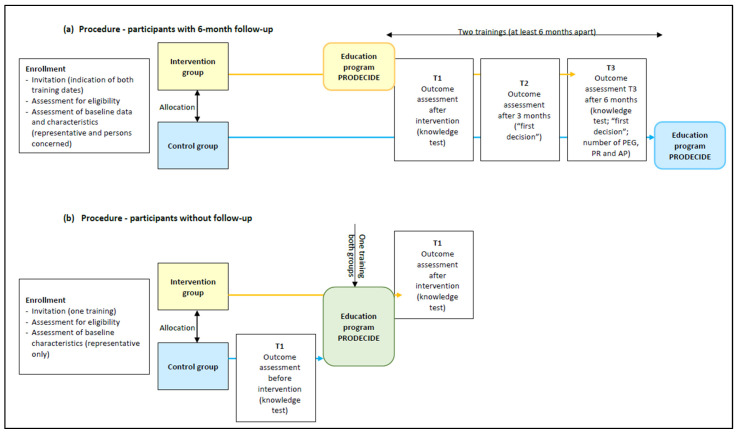
Process charts: (**a**) overview of the procedure for participants with 6-month follow-up as planned; (**b**) overview of the procedure for participants without 6-month follow-up due to difficulties in recruitment.

The PRODECIDE-RCT was a randomized controlled superiority trial with two parallel groups and a 1:1 randomization. We planned a six-month follow-up, but due to difficulties in recruitment, about 60% of participants were only included to assess the primary outcome without follow-up (see Figure 1). Additional, mixed methods were used to achieve in-depth understanding of the implementation processes. 

Before enrollment, participants provided written informed consent. The ethics committee of the German Society of Nursing Science approved the study. Details of the trial design and rationale have been reported previously [22]. 

### 2.2. Participants

Participants were professional and voluntary representatives, who represented at least one person with dementia. We also assessed data from the people with dementia (hereby referred to as persons concerned). Former participation in the PRODECIDE education program was an exclusion criterion.

The study took place in different areas of Germany. Recruitment was performed consecutively in cooperation with institutions that offer training for professional or voluntary representatives, especially with the Institute for Innovation and Practice Transfer in legal representation (Institut für Innovation und Praxistransfer in der Betreuung (ipb)), organized by the biggest German association of professional representatives [23]. Institutional cooperation included recruitment (e.g., invitations using the e-mail lists) and offering the education program. Potential participants were registered at the study center, screened for eligibility and received informed consent forms.

### 2.3. Interventions

#### 2.3.1. Intervention Group

The intervention comprised a ten-hour education program of four modules given over a period of two or three days. Module A addresses the decision-making process and introduces the assessment of harms and benefits. The aim is to enhance critical analysis of medical interventions and competencies in informed decision-making. Modules B, C and D transmit evidence-based knowledge to the example decisions. The two authors (TR, JL) conducted the face-to-face training sessions. Courses were offered free of charge or for a reduced fee for room rental and catering. Find more information in Supplement S1. The development and piloting are reported elsewhere [20].

#### 2.3.2. Control Group

As no equivalent intervention was available, the control group (CG) received standard care (usual training offers and support). After data collection was completed, CG participants were invited to take part in the education program.

### 2.4. Outcomes 

The primary outcome measure was knowledge, which was operationalized as an understanding of decision-making processes in healthcare affairs and in setting realistic expectations regarding probabilities of benefits and harms of a PEG, PRs and APs to people with dementia. The value of the primary outcome is the proportion of correct answers to 13 multiple-choice questions. The knowledge test was developed in parallel with the education program and initially tested for comprehensibility in the target group using a qualitative design. Then, it was pretested with a before–after design to roughly estimate the expected intervention effect. It was tested with a total of 34 legal representatives in five training sessions and revised iteratively.

Secondary outcome measures were (1) sufficient knowledge (using a cutoff of 70% correct answers in the knowledge test, defined by the project team members based on their scientific and empirical experiences); (2) sustainable knowledge (measured at 6-month follow-up); (3) percentage of persons concerned affected by a PEG, PRs or APs during follow-up of 6 months; and (4) result and timing of the first decision after intervention regarding a PEG, PRs and APs for the persons concerned.

In deviation from the study protocol, secondary outcomes 2 to 4 were not recorded for all of the participants. During the recruitment, we recognized that legal representatives refused to participate because the effort for data collection (especially of persons concerned) seemed too great to them or the random allocation to a training session six months later was too uncertain. Therefore, we offered shared training sessions for the intervention and control groups without follow-up assessments.

### 2.5. Data Collection

At baseline (T0), we assessed characteristics of legal representatives and baseline data of persons concerned. All data from the persons concerned were provided for the study only under an assigned pseudonym. 

At T1 (at the end of/up to 2 weeks after the intervention), the primary outcome knowledge was assessed. The questionnaire was developed and pretested with a before-after design [20,22]. It comprises thirteen multiple choice items (four choices, with only one correct answer each) on the understanding of decision-making processes (two items), on quality and validity of study results (two items) and on realistic expectations regarding probabilities of benefits and harms of a PEG, PRs and APs to people with dementia (three items per intervention). Find the questionnaire in Supplement S2. Questions with more than one answer and unanswered questions counted as a wrong answer. No summarizing score and no value of sufficient knowledge was given if four or more of the thirteen questions remained unanswered. Legal representatives in the intervention group (IG) received the test at the end of the education program. Participants in the CG with 6-month follow-up received the test by mail at the same time. To assess only T1, participants in the CG filled in the test before taking part in the education program.

We contacted the participants included for the 6-month follow-up three (T2) and six months (T3) after intervention to assess the results of the first decisions regarding a PEG, PRs and APs for the persons concerned. 

At T3, sustainable knowledge was assessed using the same test as at T1. Additionally, we assessed the number of PEG, PR and AP interventions in the persons concerned.

### 2.6. Sample Size

The assessment instruments were pre-tested with a before–after design to roughly estimate the expected intervention effects in the primary outcome of knowledge (given as a percentage of correct answers). 

A common standard deviation of σ = 0.17 was assumed in IG and CG. A mean difference of 0.085 between IG and CG can be detected by a power of 90% by the two-sided *t*-test, using a significance level of 5% based on a sample size of 86 per group (172 overall). Taking into consideration a maximum dropout rate of 20%, an overall sample size of 216 was planned. It corresponds to a medium effect of 0.5*σ, as suggested by Cohen [24]. 

### 2.7. Randomization and Blinding

After supplying their written informed consent and baseline data, participants were allocated to IG or CG and stratified by professionals and volunteers. Randomization was performed by randomly selected block sizes of four and six. An independent person performed the allocation with a computer-generated randomization list. Only data entry and analyses were blinded.

### 2.8. Data Analysis 

Data were entered into a SPSS database and double-checked by student assistants blinded to the group allocation. All quantitative data were analyzed using the intention-to-treat principle. All statistical tests were two-sided using a significance level of 5%. Baseline parameters are described by frequency tables, means and standard deviations.

The primary outcome is the percentage of correct answers for the knowledge test at T1. The values from the IG and CG were compared with adjustment for stratified randomization by professionals and volunteers using bifactorial analysis of variance (linear model with dependent variable “percentage of correct answers” and independent variables group IG/CG and professional/voluntary representatives). Professional status was chosen as independent variable following theoretical reasoning (groups differ in terms of educational background and work experience). The assumption of normal distribution was investigated by graphical methods. Furthermore, interactions between intervention and professional status were included in a secondary model. Missing values of the primary outcome were imputed by the overall mean (both groups together) of percentage of correct answers at T1. Missing knowledge at T3 was imputed in the same way but only for the invited T3 representatives. The secondary outcome of sufficient knowledge (binary, at least 70% of correct answers which means ≥ 10 of 13) at T1 was compared between IG and CG using frequency tables including Fisher’s exact test and bivariate logistic regression, including IG/CG and professional status as independent variables. No missing values of this dichotomized score were imputed, but by score definition up to 3 missing values in single items were allowed in the score, which means imputation of “wrong answers”. The interactions between intervention and professional status could not be investigated because no voluntary representative in the control group reached sufficient knowledge. For the sustainability of knowledge, the time course of the knowledge between T1 and T3 was analyzed by fitting a linear mixed model: the dependent variable was knowledge (% of correct answers), and the independent variables are IG/CG, time (T1, T3), interaction between intervention and time and professional status. To adjust for repeated measurements of the legal representatives, random effects were included (covariance pattern model with covariance structure compound symmetry). A further model including an additional interaction between intervention and professional status was fitted but not shown in detail because of the low sample size in T3.

Further secondary outcomes (the numbers of persons concerned and the percentage of these persons affected by a PEG, PRs or APs as well as the results of the first decision about a PEG, PRs or APs by the legal representative after T0) were analyzed descriptively due to the small number of participants. 

A per protocol analysis was performed without imputation of missing values in the primary outcome and after exclusion of a legal representative who was randomized in the wrong stratum (volunteer randomized in the stratum of professionals).

### 2.9. Process Evaluation and a Concept for E-Learning Modules

#### 2.9.1. Process Evaluation

Mixed methods were applied [25] according to the MRC guidance for process evaluation of complex interventions [26]. The description of the intervention and the implementation process follows the TIDieR criteria [27]. Structured documentation was used to assess data of recruitment, intervention fidelity and contextual factors. Feasibility and acceptance of the education program were assessed at the end of each training session (short verbal and structured written feedback). Coordinators from the cooperating institutions gave feedback on barriers and facilitators of implementation. In phone interviews with participants of the IG (3–6 months after training), we explored the use of educational contents in daily routines. We asked participants to describe a decision-making process, the roles of persons involved and their perceived changes in decision-making. We performed a structured documentation of the interviews. We analyzed the data in accordance with the method of collection in an iterative process [25,26]. Descriptive statistics were used for quantitative data. For qualitative data, qualitative content analyses were performed [28].

For the future implementation of PRODECIDE, quality standards were derived from existing standards in the field of further education [29,30] and optimized in the course of the study.

#### 2.9.2. Development of an E-Learning Concept

We developed different concepts for offering the education program in a blended or e-learning format. As an example, the content of module B—artificial nutrition via a PEG—was structured according to the four phases of the model of Salomon [31], transferred into the learning management system (OpenOlat [32]) and tested in a qualitative approach. The e-learning format should address both professional and voluntary representatives. Because of anticipated comparability, we also included potential volunteers, namely lay persons currently not involved in third-party healthcare decisions, into the qualitative study. To explore and understand usability problems, a concurrent think aloud method was applied [33,34]. Additionally, semi-structured interviews were performed to better understand any problems encountered and to ask for suggestions for improvement. Records of observations and interviews were summarized. Data were paraphrased and, based on usability criteria [35], categorized and interpreted. Testing and revision were performed in an iterative process to achieve data saturation.

## 3. Results

**Figure 2:**
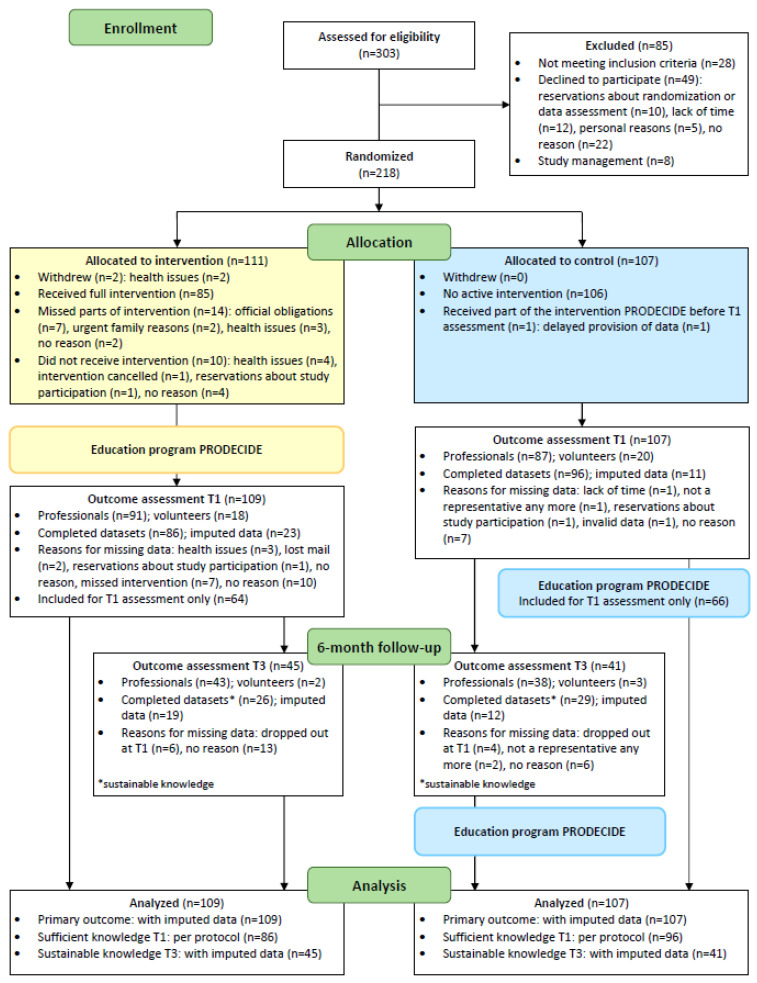
Flow diagram.

Recruitment was performed between June 2017 and June 2019. Sixteen institutions agreed to cooperate and to invite legal representatives to participate in the education program. We conducted 26 training sessions with an average of 8 participants (range 2–16). Up to August 2018, participants were included for the 6-month follow-up (*n* = 86). Due to recruitment problems, the other participants were only included for the T1 assessment. Find more information in the process charts (Figure 1). In total, 303 legal representatives were assessed for eligibility, 218 were allocated to the intervention group (*n* = 111) and to the control group (*n* = 107) and 216 could be included in the primary analysis (two withdrew informed consent including data sharing due to health issues; see Figure 2). Only a small number of voluntary representatives participated (*n* = 38). Baseline characteristics are comparable between the intervention and control groups (Table 1).

### 3.1. Primary Outcome

In the primary analysis (*n* = 216 after imputation), the percentage of correct answers in the knowledge test was 25.6% (CI 95%, 21.3 to 29.8; *p* < 0.001) higher in the intervention group compared to in the control group (Table 2). In the comparison of professional and voluntary representatives, professionals had 13.6% (CI 95%, 8.0 to 19.2; *p* < 0.001) more correct answers. The differences between groups were confirmed in the per protocol analysis. The interaction between intervention and professional status is not significant (−2.4; CI 95%, −13.6 to 8.8; *p* = 0.678). The intervention was effective regardless of the professional status, but the basic knowledge level of professionals was higher (Table 2). In T1, 21% of the knowledge tests in the intervention group had a missing value compared to 10% in the control group. The difference is statistically significant (*p* = 0.039). Group-specific histograms of percentages of correct answers did not show serious deviations from the assumption of normal distribution (not shown).

### 3.2. Secondary Outcomes

Sufficient knowledge: At T1, 51.2% (*n* = 86; CI 95%, 40.1 to 62.1; *p* < 0.001) of the participants in the intervention group had sufficient knowledge (≥10 out of 13 correct answers) and 2.1% (*n* = 96; CI 95%, 0.3 to 7.3) in the control group (RR 24.6; CI 95%, 6.1 to 98.3). Separated for volunteers and professionals, it was 38.5% vs. 0% and 53.4% vs. 2.6%, respectively. The odds ratios estimated in the bivariate logistic regression model (*n* = 182) were 49.1 (CI 95%, 11.4 to 212.6) for the IG versus the CG and 2.0 (CI 95%, 0.6 to 6.5) for professionals versus volunteers. 

Sustainable knowledge: A total of 86 out of 216 participants (39.8%) were included into the study to take part in the 6-month follow-up, and 55 provided data on this outcome (only 25%). The number of participants assessed only at T1 and the number of dropouts were comparable in both the intervention and control groups. Only five volunteers were included for the T3 assessment (three with data). In the secondary analysis, the difference between the IG and the CG for the percentage of correct answers in the knowledge test was 15.8% (CI 95%, 9.7 to 22.0; *p* < 0.001), which was slightly smaller than in T1 (estimated by the linear mixed model). 

Percentage of persons concerned affected by a PEG, PRs or APs: Due to the changes in recruitment, only 34% of the included representatives (37% in the IG and 32% in the CG) provided data on the people with dementia (persons concerned). Only three volunteers provided data. Overall, 74 representatives provided data from 329 persons concerned (55.2% female; age: 25.6% ≤ 69 years, 29.6% 70–79 years, 34.9% 80–89 years and 10.0% ≥ 90 years old). Only 43 representatives provided data on the persons concerned at both time points, at T0 and at 6-month follow-up (T3). These data are presented in Table 3. There seems to be a tendency for a reduction in APs in the IG (56.7% to 40.7%) without any causal conclusions being drawn from this. There is no hint for differences between the groups for PRs or a PEG. 

First decision regarding a PEG, PRs and APs for the persons concerned: Thirty-three representatives reported at least one decision (thirteen in the control and twenty in the intervention group). Eleven participants reported a decision on PRs, fifteen on APs and twenty-one on a PEG. Due to the small number, we performed no further analyses.

### 3.3. Process Evaluation and a Concept for E-Leraning Modules

A detailed presentation of the results can be found in Supplement S1.

#### 3.3.1. Influence of the PRODECIDE Education Program on Decision-Making Processes

In the interviews, participants of the IG reported that they have used the knowledge and the educational materials to inform decisions on a PEG, PRs or APs. In their self-assessment, they weighed up the options more critically. But they also stated that it was difficult to put the knowledge into practice. A self-perceived barrier for the implementation of training contents was that other persons involved in the decision-making process (e.g., physicians) lacked the willingness to discuss options. Further barriers seem to be the lack of time, traditional role models and the trust in the physicians’ decisions.

#### 3.3.2. Barriers and Facilitators for Implementation

Recruitment was a major problem. Barriers at the institutional level were a lack of resources to organize and provide training or a lack of awareness of one’s own responsibility and of the need to offer training. At the individual level, we had insufficient access to non-participants to assess reasons systematically, but we assumed that existing structures and the usual offers in the region mattered. Persons registered for the training but who missed parts or the entire training, gave as their reasons health issues, workload or official obligations like appointments at court. Recruitment would probably be easier without study conditions. The additional requirements have made participation on an institutional as well as an individual level even more difficult.

Feedback from participants attending the training was mostly positive. Participants were especially interested in the use of antipsychotics for people with dementia, in psychotropic medications in general and in chemical restraints. Coordinators from the cooperating institutions also rated the contents of the training as relevant. Overall, an implementation seems possible.

#### 3.3.3. Concept for E-Learning Modules

The results of the pilot test of the e-learning module provide important aspects that will be taken into consideration in the further development of an e-learning concept. The usability test showed that a stringent menu navigation and structured learning sequences should be applied. A flexible offer in a modular blended learning format seems to be appropriate.

## 4. Discussion

We evaluated the efficacy of the PRODECIDE education program in terms of increasing knowledge for voluntary and professional representatives. Overall, the IG received 25.6% more correct answers in the knowledge test than the CG. Thus, the education program was equally effective for volunteers and professionals. However, the level of knowledge was considerably lower among the volunteers (58.8% IG, 31.3% CG) than among the professionals with 71.2% correct answers in the IG vs. 46% in the CG. Neither professionals (2.6%) nor volunteers (0%) showed sufficient knowledge in the CG. Even in the intervention group, after participating in the education program, only 53.4% of the professionals and 38.5% of the volunteers achieved sufficient knowledge. These numbers are alarmingly low, especially in view of the responsibilities in health care, and emphasize the importance of optimizing the system of legal representation. Legal representatives should protect the autonomy of the people concerned as best as possible, including in claiming the vulnerable group’s right to evidence-based, informed decisions, with the aim of reducing medically and ethically questionable decisions and intervention. For this, basic knowledge in all the three disciplines—medicine, law and ethics—is essential. Therefore, only the linking of these disciplines in the application will lead to an improvement in the decision-making processes. Knowledge alone is not enough for practical implementation, but it is an initial prerequisite. Regarding the low and insufficient knowledge, there are several aspects to be discussed. One is the low proportion of legal representatives with sufficient knowledge. The knowledge test was developed and pre-tested in the preliminary project [16]. The project team members defined relevant knowledge questions and the cutoff for sufficient knowledge, based on their scientific and empirical experiences. No comparable test is known. The pre-test showed no evidence of a ground effect. Nonetheless, the definition of sufficient knowledge is a subjective one. 

An understanding of the decision-making process and realistic expectations about the benefits and harms of the medical measures are prerequisites for informed and evidence-based decision-making [36]. Therefore, we hypothesized that sufficient knowledge enables legal representatives to make evidence-based decisions, which would lead to a reduction in PRs, PEGs and APs. 

Due to a lack of data, we cannot make a statement on the implementation of the knowledge acquired. The reported numbers of PEGs, PRs and APs roughly correspond to the prevalence in German nursing homes (5% for PEG [37], 12.5% for PRs [37] and almost 30% for APs [38]). Apparently, the cohort analyzed closely mirrors the real situation. No differences between the IG and the CG could be assessed.

The process evaluation indicates that participants are motivated to apply the new knowledge, but the barriers in the decision-making process are still very high. Medicine and law are disciplines that are generally viewed with great respect. The demand that legal representatives act as decision-makers at the intersection of these two disciplines is challenging and probably needs more than knowledge input. In addition to the barriers mentioned, such as difficult discussions with doctors, unconscious, emotional or value-based processes may also play a role. For example, PRs may be approved due to a desire for safety, although contrary evidence is found from the training. Presumably, making decisions based on objective criteria is particularly difficult for voluntary representatives who have an emotional relationship with the person concerned. A further aspect that requires a detailed discussion is the noticeable difference in knowledge between volunteers and professionals. However, the comparison should be interpreted carefully, as the group of volunteers was significantly smaller (38 volunteers versus 178 professionals). Volunteers were harder to reach, although they represent the larger group [4]. This group is subdivided into people who do voluntary work for others and into family members (around 85%), who were appointed as legal representative out of necessity [39]. It is likely that there is also a difference in knowledge between these groups. Participation in the study was more likely to come from non-family caregivers since they are to a greater extent active in associations for voluntary representatives.

The German guardianship court regulates the legal representation. The legislature has deliberately given “…priority to voluntary representatives over professionals. This requires not only consideration of the interests of the state treasury […], but also the need to reserve professional representatives with special qualifications for those affected, who really need the corresponding knowledge and skills of the representative” [3]. It is therefore accepted that voluntary representatives have less knowledge. 

A common assumption is that family members as legal representatives nevertheless make appropriate decisions because they know the persons concerned and their life circumstances and wishes very well [20,40]. However, a systematic review has shown that patient-designated and next-of-kin surrogates incorrectly predict patients’ preferences in one third of cases concerning end-of-life treatment decisions for incapacitated patients [41]. On the one hand, this shows how important it is to take timely precautions in the form of advanced care planning and advanced directives and, on the other hand, that it would be appropriate to expect voluntary representatives to have the same knowledge on decision-making processes as that of professionals. For voluntaries, however, the scope would have to be adapted, but, even with a less time-consuming offer, there might be difficulties in motivating this group to participate. 

Professionals generally have a high level of education. We found considerable differences in the underlying education and training between volunteers and professionals. Twenty-eight percent of the volunteers had a university degree compared to eighty-two percent of the professionals. However, from our point of view, the mere fact that a university degree exists does not confirm whether specific knowledge is sufficiently available for acting as a legal representative.

The qualification of professional representatives has been politically discussed in Germany for several years. In a large survey in 2015, 80% of them rated their knowledge of healthcare as at least good [42], but results of an external assessment and results for volunteers are not available. 

In May 2021, the German “Betreuungsrecht”, which is to be understood in an international context analogous to the law of guardianship/custodianship, has been reformed, and the implementation of a legal basis by legal representatives—both volunteers and professionals—is mandatory from 1st January 2023. A significant change is the strengthening of autonomy as the highest ethical principle, based on the Convention on the Rights of Persons with Disabilities. Unfortunately, another law, the Patients’ Rights Act, which regulates a right to evidence-based and appropriate information on health issues, was not taken into account in the formulation of future training content.

The new legislation is re-regulating the qualification of professional representatives [43]. A formal registration procedure is to be introduced to ensure a uniform quality. Professional representatives must prove personal and professional minimum qualification requirements. The qualification is assessed according to the original profession, but further qualification is expected in the areas that are not covered by this. For the majority of professional representatives, this affects the health sector. Nevertheless, it is to be feared that there still will only be few qualification offers in healthcare, possibly just because of good self-assessments in the survey made in 2015 [42]. This would contradict both, our results—nearly none of the participants in the CG had sufficient knowledge—and how informed decision-making is a key issue in the legal representation of people with dementia and other target groups as well. New legislation is also regulating how voluntary representatives are required to enter into an agreement with a “Betreuungsverein” (association for voluntary legal representatives), which arranges support and guidance. This agreement also regulates, among other things, how representatives take part in introductory and advanced training events. It is unclear to what extent voluntary representatives enter into and comply with these agreements, and whether there will be consequences if they do not. This question arises in particular given the fact that there are already too few voluntary representatives and that, for financial reasons, the dedication of volunteers is—from a political point of view—still desirable.

The process evaluation has shown that the training leads to an increase in knowledge, but there are still a number of barriers for implementation. The role of the legal representative in a decision-making process is unclear and a paternalistic view prevails. Representatives do not have the courage to represent newly acquired knowledge to doctors or nurses. 

A role-play in which training participants could practice acting as confident and equal discussion partners in a decision-making process was found to be particularly helpful. In the future, additional training modules for doctors or nurses on the subject of evidence-based (proxy) health decisions would be desirable.

### Strengths and Limitations

This study was rigorously planned and carried out in accordance with the UK MRC framework for the development and evaluation of complex interventions [44]. The intervention—the education program—as well as the evaluation instruments were developed and pilot-tested in a preliminary study [20]. The recruitment of the participants was challenging, despite close cooperation with acknowledged institutions. The random allocation to the intervention or control groups and the length of time before training took place (in the control group) reduced the acceptance of the study as well as the questionnaires at several measurement points. As a result, the desired number of participants for the secondary outcomes could not be achieved. For the primary outcome, enough participants could only be recruited by changing the recruitment process. We continued to recruit and randomize consecutively, but, in one training session, participants from both groups took part and only data for the primary outcome were collected. Due to the nature of the intervention, participants and researchers conducting the education program and data collection were not blinded. A structure-identical intervention could not be offered. However, blinding was performed during data entry and analysis. For the primary outcome, there were about twice as many missing values in the knowledge test in T1 in the IG as in the CG (*p* = 0.0391). A possible explanation is that the participants in the control group had to provide the filled in tests before attending the training. In contrast, participants in the intervention group were asked to send the test in the week after the training per mail or fax. Despite sending reminders, not everyone followed this instruction. Therefore, a bias of the results cannot be ruled out. Another reason could be that people who would not be able to answer the questions might not deal with it at all. Results on sustainable knowledge at T3 are strongly limited by low sample size and non-random selection of a subgroup of responders after 6 months.

## 5. Conclusions

The PRODECIDE education program can improve the knowledge of legal representatives and thus contribute to better qualification in a key issue for this profession. It enables participants to assess the benefits and harm of medical measures more realistically. Still, it is not possible to draw conclusions about the implementation of knowledge in everyday working life. The process evaluation provides indications that a critical attitude is being developed, but, in practice, there are still considerable barriers to applying the knowledge. One main barrier probably could be physicians’ attitudes regarding the prescription of antipsychotics, which should also be addressed. These barriers should be addressed in further projects. The lack of standardized training for professional representatives and very low-threshold requirements for voluntary representatives are international problems. This does not correspond to the ethical claim of informed decision-making with the participation of a representative decision-maker as well [45]. Especially for volunteers, more research is needed about the scope, content and didacticism of education programs and, last but not least, about how to reach them so that they recognize the importance of the content and are able to access the training. Finding a solution to this discrepancy remains a challenge.

## Figures and Tables

**Table 1:** Baseline Characteristics.

Characteristics	Intervention (*n* = 109)	Control (*n* = 107)
Age, mean (standard deviation (SD))	52.0 (10.2)	52.5 (10.0)
Female, N (%)	73 (67.0)	81 (75.7)
Professional status:		
Professionals, N (%)	91 (83.5)	87 (81.3)
Volunteers, N (%)	18 (16.5)	20 (18.7)
No. of persons represented *, mean (SD)	29.2 (19.2)	28.3 (18.7)
Years being a legal representative ^+^, mean (SD)	9.4 (7.7)	7.9 (7.4)
University degree, N (%)	82 (75.2)	80 (74.8)
Social work/social pedagogy, N	37	35
Education/pedagogy, N	12	10
Legal studies, N	10	7
Medicine/healthcare, N	1	0
Guardianship, N	4	1
Others/no information, N	18	27
Completed vocational training, N (%)	36 (33.0)	29 (27.1)
Commercial training, N	14	10
Medicine/healthcare, N	5	8
Education/pedagogy, N	7	1
Others	10	10

* IG missings = 1; ^+^ IG missings = 2, CG missings = 1.

**Table 2:** Results of the knowledge test at T1.

	Groups	Percentages of Correct Answers * (CI 95%)	Differences between Groups (CI 95%)	*p*-Value
**Primary model**	Intervention group (*n* = 109)	69.0 (66.0 to 72.0)	25.6 (21.3 to 29.8)	*p* < 0.001
	Control group (*n* = 107)	43.4 (40.4 to 46.4)		
	**Professional status**			
	Professionals (*n* = 178)	58.7 (56.3 to 61.0)	13.6 (8.0 to 19.2)	*p* < 0.001
	Volunteers (*n* = 38)	45.1 (40.0 to 50.2)		
**Interaction model**	**Professionals**		25.2 (20.5 to 29.9)	*p* < 0.001
	Intervention group (*n* = 91)	71.2 (67.9 to 74.4)		
	Control group (*n* = 87)	46.0 (42.6 to 49.3)		
	**Volunteers**		27.5 (17.4 to 37.7)	*p* < 0.001
	Intervention group (*n* = 18)	58.8 (51.4 to 66.2)		
	Control group (*n* = 20)	31.3 (24.3 to 38.3)		

* primary analysis, including imputed data (overall mean value of 56.297549% was imputed in missing values).

**Table 3:** Percentage of persons concerned affected by a PEG, PRs or APs.

		Intervention Group (*n* = 23 AP; *n* = 22 PEG, PR)	Control Group (*n* = 20)
Percentage of persons concerned with a PEG per representative, mean (SD)	T0	8.0% (±13.3)	1.7% (±4.3)
	T3	11.9% (±24.2)	2.1% (±4.4)
Percentage of persons concerned with PRs per representative, mean (SD)	T0	15.8% (±23.3)	14.4% (±17.8)
	T3	19.1% (±29.0)	12.4% (±17.4)
Percentage of persons concerned with APs per representative, mean (SD)	T0	56.7% (±37.8)	36.6% (±30.2)
	T3	40% (±40.2)	35.1% (±34.7)

## Data Availability

The datasets analyzed during the current study are available in the SowiDataNet, a service of the GESIS—Leibniz Institute for the Social Sciences (https://data.gesis.org/sharing/, accessed on 4 March 2024): “Informed decision-making with and for people with dementia—Efficacy of the PRODECIDE education program for legal representatives: a randomized controlled trial (PRODECIDE-RCT) and detailed process evaluation” (https://doi.org/10.7802/2466, accessed on 4 March 2024).

## Supplementary Materials

The following supporting information can be downloaded at https://www.mdpi.com/article/10.3390/geriatrics9030060/s1, Supplement S1: Process evaluation and a concept for e-learning modules; Supplement S2: knowledge test.

## Trial Registration

ISRCTN17960111, Date: 01/06/2017.
