# Supplementary material for: Informed Decision-Making with and for People with Dementia—Efficacy of the PRODECIDE Education Program for Legal Representatives: A Randomized Controlled Trial (PRODECIDE-RCT) and Process Evaluation
Source: Geriatrics (Basel). 2024 May 9;9(3):60. doi: 10.3390/geriatrics9030060 (PMC11130972; doi:10.3390/geriatrics9030060)
Supplement: Supplementary file 1 [file PRODECIDE-RCT_Supplement 2_knowledge test.pdf › PRODECIDE-RCT_Supplement 2_knowledge test.pdf]

**Dear Participants,**

You are taking part in the PRODECIDE-RCT study. With this questionnaire, we want to find out what you know about health care decisions.

The questionnaire contains 13 items on healthcare decisions in people with dementia.

Please answer ALL questions. If you are unsure of your answer, choose the answer you think is most appropriate.

## Module A: Decision-making process

Item 1 and 2 deal with ways in which legal representatives might act when a health care decision is required. In the green-bordered boxes, you will find case studies. Please read the case studies carefully. The following items will relate to this.

> You were assigned 4 weeks ago as legal representative for an 81-year-old woman (Mrs. S.). The woman still lives in her own apartment. There she fell and was found helpless by the neighbour. A forearm fracture (right) was found in the hospital. In addition, the doctor in the emergency room will inform you by telephone that Mrs. S. would be beginning dement and constitute a nursing case. He informs you that the arm needs surgery in the next few days. The doctor needs a signature on the consent form. You are being asked to take action.

**Item 1. What do you do first?** (One answer is correct.)

- ☐ You ask the surgeon to inform you about the risks of the operation by telephone. Then you provide your consent to the operation.
- X You are planning a visit to the hospital to discuss the proposed procedure in a personal interview with Mrs. S.
- ☐ You are unsure whether Mrs. S. can provide consent herself. You call the neighbor to get her assessment. Only after the phone call do you decide whether to give consent.
- ☐ You suspect that Mrs. S. would consent to the operation and thereupon provide your consent to the operation by fax.



*Correct answers are highlighted blue*

## Supplement 2 – Knowledge test

…Mrs. S. 's condition is deteriorating. In addition to the forearm fracture, the fall probably also resulted in a concussion. Fr. S. doesn't know where she is or what happened. You meet the neighbour at the clinic. She reported that Mrs. S. had always placed great trust in doctors and had agreed to a similar operation last year after a similar fall. Mrs. S. had discussed the reasons for this decision at length with her neighbour.

**Item 2. What do you do next?** (One answer is correct.)

- ☐ Mrs. S. is unable to make a decision on her own. You submit an application for authorization of the operation to the supervisory court.
- X Mrs. S. cannot understand the information on the proposed procedure. Based on the testimony of the neighbor, you assume that Ms. S. wishes the operation and provide consent.
- ☐ What the neighbor says is irrelevant to you, you decide on the basis of your personal conviction that the procedure is the best solution for Mrs. S. and provide your consent.
- ☐ As Mrs. S. is unable to provide her consent, a decision must be taken for the benefit of the person concerned. You discuss this with the doctors in charge.

## Module A: Methods

Mrs. S. is feeling better again. She's doing fine in her apartment. You accompany her to a doctor's appointment at her request. She has been in neurological treatment for some time because she complains of recurring severe headaches. The neurologist wants to offer her a new drug KOPORMIN.

Item 3 and 4 deal with information on the benefits and harms of measures that should be weighed when a health care decision is required. In the green-bordered box, you will find a case study to which the items refer. Please read the case study carefully before answering.

**Item 3.** Below you will find several sources from which the neurologist may have obtained his knowledge. **What is the best source of information about the effectiveness of the drug?** (One answer is correct.)

- ☐ From own experience
- X Results of a study with intervention and control group (randomly allocated)
- ☐ Results of a cohort study
- ☐ Prescribing the drug for 4 weeks and monitoring its effectiveness

## Supplement 2 – Knowledge test

In the green-bordered box, you will find information to which the next item relate. Please read it carefully before answering.

The drug KOPORMIN has been evaluated in a scientific study.
200 participants with headaches similar to Ms. S. were studied over a period of 4 weeks. The study participants were randomly assigned to two groups:

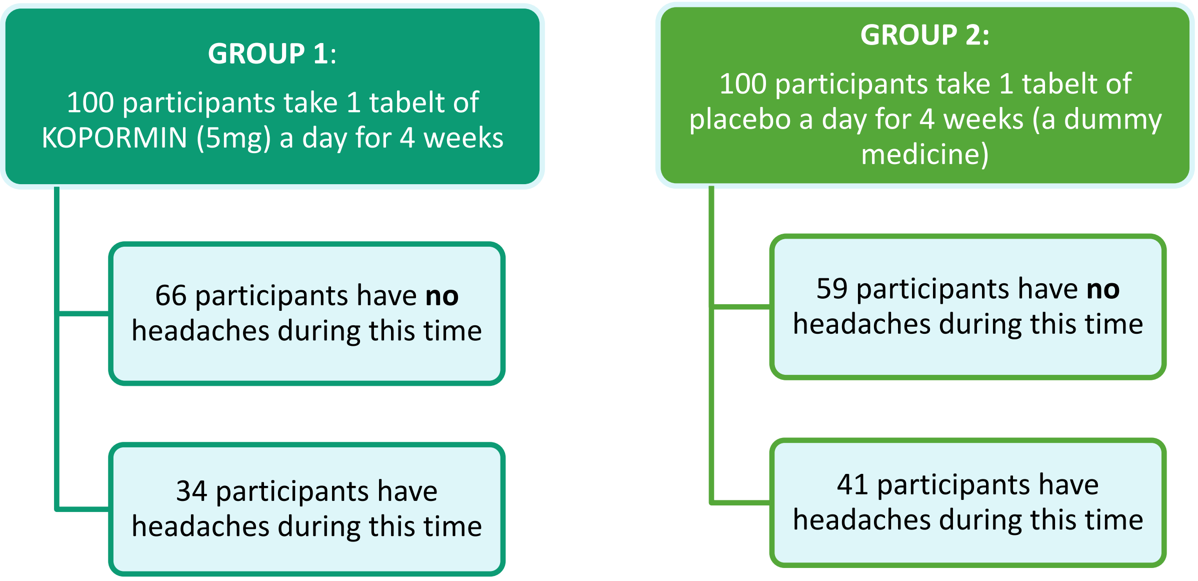


**Item 4. How many participants have a benefit <u>DUE TO</u> the use of KOPORMIN, that is, no headache within the study period due to the drug?**
(One answer is correct.)

X 7 participants
☐ 18 participants
☐ 32 participants
☐ 66 participants


*Correct answers are highlighted blue*

## Supplement 2 – Knowledge test

## Module B: Decision percutaneous endoscopic gastrostomy (PEG)

Items 5 to 7 deal with the decision for or against installing a PEG (stomach tube).

**Item 5.** Some people become a PEG because of their dementia. Objectives include longer survival, improved quality of life, improved nutritional status (weight, BMI) and the avoidance of pneumonia or pressure ulcers. Such correlations can be investigated in scientific studies. **Which statement is correct?** (One answer is correct.)

Scientific studies have shown that there is a correlation between artificial nutrition via a PEG…

X …and **more frequent** pressure ulcers.
☐ …and **improved** nutritional status.
☐ …and a **longer** survival period.
☐ …and a **poorer** quality of life.

**Item 6.** Different aspects should be taken into account when deciding whether or not to install a PEG due to dementia.
**Which statement is correct?** (One answer is correct.)

☐ The decision may be presented as urgent by doctors or nurses. In order to obtain approval by the supervisory court, an urgent application should be made.
☐ In order to reduce the risk of pneumonia, the installation of a PEG should be approved.
☐ Before consent to a PEG is provided, a nasal tube should always be tested.
X It is important whether there are other diagnoses besides dementia, such as a stroke.

**FRAGE 7.** Different persons and institutions may be involved in deciding whether or not to install a PEG due to dementia.
**Which statement is correct?** (One answer is correct.)

☐ If the legal representative makes the decision to install a PEG, it is necessary to have the decision approved by the supervisory court.
☐ The decision on whether to install a PEG must be made by the treating doctor, as it is a medical measure.
X Rejection of a PEG does not require approval by the supervisory court if the doctor and the legal representative agree that the rejection is in accordance with the wishes of the person concerned.
☐ If the doctor and the legal representative disagree on whether to install a PEG, the decision must be taken by the supervisory court.

## Supplement 2 – Knowledge test

## Module C: Decision physical restrains (PR)

Items 8 to 10 deal with the decision for or against PR.

**Item 8.** In some nursing homes, PR such as bed rails, bed straps or fixed snap tables are used on people with dementia. Whether these measures are applied more or less frequently may have different reasons. Scientific studies have tried to demonstrate which factors may be associated with the use of PR.
**Which statement is correct?** (One answer is correct.)

Scientific studies have shown that there is a correlation between the frequencies of PR…

☐ …and the staffing ratio of the nursing home.
☐ …and the frequency of falls.
X …and the attitudes of nurses to PR.
☐ …and the age of the nursing home residents.

**Item 9.** Different aspects may play a role in deciding whether or not to use PR.
**Which statement is correct?** (One answer is correct.)

☐ The decision to use PR may be necessary because a nursing home is unable to implement alternatives.
☐ There is evidence that the quality of life of nursing home residents improve when fewer PR are used.
☐ The reduction of PR in nursing homes leads to increased falls among residents.
X Many alternatives to avoid PR are proposed, but most have not yet been shown to have a benefit.

**Item 10.** Different persons and institutions may be involved in the decision for or against the use of PR.
**Which statement is correct?** (One answer is correct.)

☐ In the case of uncertainty as to whether or not to apply PR, the decision is left to the supervisory court.
☐ The use of a split or half bed rail requires the approval of the supervisory court.
☐ If PR are necessary to carry out medical orders, there is no need for authorization from the supervisory court, only a doctor’s prescription.
X If a person with severe dementia makes only involuntary movements, i.e., not with the will to get up, a bed rail does not have to be approved by the care court.



*Correct answers are highlighted blue*

## Module D: Decision antipsychotic drugs (AP)

Items 11 to 13 deal with the decision for or against prescribing antipsychotics.

**Item 11.** Some people get prescribed antipsychotics (also called neuroleptics) due to their dementia. When prescribing medicines, doctors can follow so-called medical guidelines (treatment recommendations). There is a German-language guideline for the treatment of dementia.
**Which statement is correct?** (One answer is correct.)

☐ Antipsychotics should be used on a trial basis regardless of the cause of challenging behavior.
X People with dementia should be treated with antipsychotics for as short a period as possible.
☐ As the efficacy of alternative treatment measures has not been sufficiently demonstrated, they are not recommended in the guideline.
☐ Antipsychotics should only be used in people with dementia if treatment with antidementia has been unsuccessful.

**Item 12.** The efficacy of risperidone (tradename Risperdal, atypical antipsychotic) in the treatment of challenging behaviors has been evaluated in randomized controlled trials. In addition to efficacy, possible side effects were also investigated.
**Which statement is correct?** (One answer is correct.)

☐ Less than 10 in 100 people with dementia experience incontinence when treated with risperidone.
X More than 10 out of 100 people with dementia experience movement disorders due to treatment with risperidone (2mg/day).
☐ Less than 1 in 100 people with dementia have an additional stroke when treated with risperidone.
☐ More than 30 out of 100 people with dementia experience somnolence and fatigue when treated with risperidone (2mg/day).

**Item 13.** Different aspects should be taken into account when deciding whether or not to prescribe an antipsychotic drug.

**Which statement is correct?** (One answer is correct.)

- ☐ Prescribing an antipsychotic drug to the night is appropriate for people with dementia to make it easier for them to respect night's sleep.
- ☐ Treatment of challenging behavior, e.g., with risperidone, provides a significant improvement in daily care.
- X If antipsychotics are required to treat agitated and aggressive behavior, risperidone should be preferred.
- ☐ Treatment with an antipsychotic drug leads to improved cognitive performance in people with dementia.

## Thank you very much for your participation!
