# Supplementary material for: Informed Decision-Making with and for People with Dementia—Efficacy of the PRODECIDE Education Program for Legal Representatives: A Randomized Controlled Trial (PRODECIDE-RCT) and Process Evaluation
Source: Geriatrics (Basel). 2024 May 9;9(3):60. doi: 10.3390/geriatrics9030060 (PMC11130972; doi:10.3390/geriatrics9030060)
Supplement: Supplementary file 1 [file geriatrics-09-00060-s001.zip › PRODECIDE-RCT_Supplement 1_process evaluation.pdf]

Supplement 1

Process evaluation and a concept for E-learning modules

Process evaluation

Description of the intervention

The description of the intervention and the implementation process follows the TIDieR criteria [27]. The PRODECIDE education program was developed in 2014 [20]. The educational content is based on systematic reviews of the evidence and on the results of interviews with legal representatives and senior citizens about their experiences, preferences and wishes regarding decision-making processes in healthcare. More on the curriculum and the development process is reported elsewhere [20].

The ten-hour education program comprises four modules. Module A addresses the decision-making process and introduces study designs as well as the assessment of harms and benefits. The aim is to enhance critical analysis of medical interventions and competencies in informed decision-making. The modules B, C and D transmit evidence-based knowledge to the decisions given as examples (artificial nutrition via a percutaneous endoscopic gastrostomy (PEG), the use of physical restrains (PRs), and the prescription of antipsychotic (AP) drugs). Due to the methodical approach, transferability to other decisions in healthcare is anticipated.

The participants received written education material and evidence-based information leaflets. These materials as well as the presented sources of information (e.g. guidelines and decision aids) should support further decisions. The sessions comprise presentations, discussions and group work. The discussion of case studies shows the practical relevance of the educational content.

The education program is primarily designed for small groups of up to 25 people. The ten hours are given over two or three days. There are no special requirements regarding the location but a beamer, screen and a pin board are helpful.

The two authors (TR, JL) conducted the face-to-face training sessions. They did this mostly together due to the additional study documentation and assessment. TR is a medical doctor, health scientist and professional representative. JL is a nurse and health scientist.

Implementation process

To facilitate implementation and recruitment, we offered the intervention in cooperation with institutions responsible for the training of professional or voluntary representatives. In total, we invited 71 institutions (31 regional departments, 33 associations responsible for legal representatives and 7 education institutions) from all over Germany. We contacted the institutions by mail and phone and 16 agreed to cooperate and to organize training sessions. Some of the addressed institutions do not have the resources; others may not feel responsible or see no need to offer trainings regularly. From several institutions, we received no response. In cooperation with the institutions, we scheduled 41 training sessions, which were offered free of charge or for a reduced fee for room rental and catering. We had to cancel 17 sessions due to low registration numbers. Registration numbers varied widely between different regions, even with the same institution offering the training at various places. We had insufficient access to non-participants to assess reasons systematically, but we assume that existing structures and the usual regional offers were the cause.

The main cooperation partner ipb provided 12 out of the 26 training sessions. We offered 2 sessions at the University of Hamburg and 12 in cooperation with other institutions. The 26 training courses took part with between two and 16 participants (mean 8). The ipb only offers training for professional representatives. That is one reason why the number of included

volunteers (n=38) is low. In general, it is difficult to get access to the major group of family members, even by associations specialized in the support of voluntary representatives.

Recruitment was performed between June 2017 and June 2019. The cooperating institutions distributed the invitations for the training sessions. Therefore, we have no data to provide response rates. We assume they were low, especially in the first months of recruitment. In total, 303 legal representatives were assessed for eligibility and 216 could be included in the primary analysis. Up to August 2018, participants were included for the 6-month follow-up. Therefore, two sessions were scheduled at each location and the participants had to agree to be randomized either to the first training (intervention group) or to the second training after six months (control group). This procedure was not well understood and the willingness to agree to it was low. To achieve at least the calculated sample size for the primary outcome we changed the procedure, provided only a single training session and assessed only baseline characteristics of legal representatives and the primary outcome. From June 2017 up to August 2018, we included only 86 persons for the 6-months follow-up and for the following ten months, we were able to include 130 persons.

Intervention fidelity and adaptions

The 26 training sessions took part in different regions of Germany (Hamburg, Bremen, Schleswig-Holstein, Saxony-Anhalt, North Rhine-Westphalia, Lower Saxony, Bavaria, Saxony, Baden-Württemberg, and Mecklenburg Western Pomerania). Locations and equipment varied widely. Some cooperating institutions provided professionally equipped seminar rooms; others required some improvisation to use the different media. Most of the sessions took part on two consecutive days (first day from 1:00 pm to 6.00 pm, second day from 9:00 am to 2:00 pm). We also offered three single appointments of about three hours each for voluntary representatives.

Participants in the intervention and control groups attended the training sessions together. In total, 245 persons were registered for the training (216 study participants, 29 other persons, e.g. members of the cooperating institutions), but only 219 persons took part. Reasons given for missing parts of or the entire training were health issues, workload or official obligations like appointments at court.

The timetable of the training is tight. Delays were caused by organizational difficulties (e.g. seminar room was not prepared), study administration (e.g. knowledge test not completed in in advance, questions about the methodical approach) or by participants coming late or leaving earlier. Especially in one training session, participants were coming and going nearly all the time. This was accompanied by critical discussions regarding the responsibilities of legal representatives and the legitimacy of questioning physicians' medical decisions. Most of the participants hold a paternalistic view and some doubted the presented evidence (e.g. the lacking benefit of tube feeding in dementia). This group represented various cultural backgrounds. The cultural background may influence the acceptance of the training.

Discussions emerged in all of the training sessions, at different time points and on various themes. Most of them were constructive and showed interest. The exchange of different views and experiences is an important part of the training.

Especially in the first module, discussions arose on the factors influencing decisions and the decision-making concepts. In addition, to handle the first task, some participants needed more explanation than scheduled. Therefore, in most sessions the first module exceeded the intended time. The module on antipsychotic medication often also took more time than intended. It is complex but most participants also rated it as very important.

Due to the delays, there was often not enough time to discuss the second case example on physical restraints and antipsychotic medication in detail. There was only a short presentation or a recommendation to discuss it later with colleagues.

In addition, but not affecting the intervention itself, the lack of time leads to a systematic difference in the response rate between the intervention and control groups. Participants in the control group had to submit the knowledge test before the training session. Participants in the intervention group were often not able to submit the test directly after the training. Despite the request to send it back to the study center in the next few days and repeated reminders, some participants did not submit it at all.

The training is standardized. Except for one session, the modules were offered in the same order. We had developed moderation cards in the piloting phase but as the routine improved, some changes in the training were established. The case example on tube feeding was not only discussed but also performed as role-play. Participants had to defend different point of views, not only their own. To make the first task on factors influencing decisions easier, we presented headlines to cluster the factors. A further working task was shortened to save time, if necessary.

We integrated new study results on psychosocial interventions to reduce physical restraints and antipsychotics into the presentation. In addition, we provided current information and publications to interested participants.

Participants responses to the intervention

The participants were invited to give a short verbal and written feedback at the end of the training session. The verbal feedback on the training was mostly positive. It was rated as informative, practical, worthwhile despite long years of experiences and as a reason to think more critically.

In the written feedback, 5-point likert-scales were included (results see Table S1). In addition, an open-ended question asked for the most important contents of the training. The participants mentioned all main medical but also legal aspects. Participants were especially interested in the use of antipsychotics in people with dementia, in psychotropic medications in general and in chemical restraints. They rated the structured presentation of decision-making concepts and processes as helpful, although some participants knew the concepts already. The parts on the assessment of harm and benefit and on study designs were rated as important and interesting but also as too long and too scientific. Participants liked the training because of the small groups, working tasks, praxis examples, the role-play, discussions, learning materials, hints for further information and the coincidence of high practical relevance and high professional standard. They criticized the lack of time and, therefore, that some contents or discussions were too detailed and too long.

Locations and catering seem to be important factors. The feedback here varied widely, depending on the offers of the cooperating institutions. Some critical comments were directed at the study administration and the methods of data collection. The aim of the assessment was not clear enough to all participants or the multiple-choice questions did not appear suitable.

Table S1. Written feedback

Items	Ratings (range 1-5), mean
How do you rate the training regarding…	
meeting your expectations	1.6
practical relevance	1.5
amount of content	1.5
utility	1.5
pace of work	1.7
learning materials	1.2
How do you rate the teacher regarding…	
understandability	1.2
professional expertise	1.2
moderating the group and discussions	1.4
Ratings: 1 = very good; 2 = good; 3 = satisfying; 4 = sufficient; 5 = insufficient<br>Number of participants n=161	

Impact on decision-making processes

We asked participants for the result and timing of the first decision after intervention regarding PEG, PR and AP for the persons concerned. Participants in the IG were also asked for an interview on the decision-making process and the implementation of training contents. Thirty-three representatives reported at least one decision (13 in the control and 20 in the intervention group). Eleven participants reported a decision on PR, 15 on AP and 21 on PEG. Regarding the incidences, decisions on AP should have been the most frequent decision. These results give a hint that decisions on a PEG are more recognizable or that representatives are more involved in it, or both.

Occasions for the decisions were factors such as a change or occurrence of symptoms, new diagnosis, quality of life, age, difficult care situations, wishes of the person concerned, or requests of nurses, relatives or physicians. Additionally, the involvement of the court was mentioned. Some participants of the IG also reported that they had reviewed the medication plans actively after attending the training. The decisions were influenced by the health status

of the person concerned, the care situation, agreements with other persons involved, and the presumed will or the living will.

The reports on decision-making processes were comparable to reports assessed in former interviews with representatives not attending the PRODECIDE program [20]. Nevertheless, participants reported that they have used the knowledge and the educational materials to inform the decision. In their self-assessment, they weighed up the options more critically. However, they stated that it was difficult to put the knowledge into practice. Self-perceived barriers for the implementation of training contents ranged from the involvement of other persons in the decision-making process (e.g. physicians) who lacked the willingness to discuss options, up to threats to terminate the care. Some care situations seem to be too difficult to implement alternative interventions. Participants also reported time issues to deal with some matters, e.g. with the medication prescriptions. Further barriers seem to be the traditional role models and the trust in the physicians' decisions.

To gain further insight into behavioral changes, intermediate outcomes (e.g. number and content of conversations with healthcare professionals) ought to be assessed in a random sample of participants. Due to the small number of participants with 6-months follow-up, these were not assessed.

Barriers and facilitators for the implementation of the PRODECIDE education program

Reasons for participation were professional interest, especially in new contents, networking and exchange as well as (local) requirements to attend trainings regularly.

Participants would like to attend further trainings in order to work on the themes more deeply (e.g. alternatives for restraints) and they would be interested in more aspects of dementia (e.g. handling people with dementia), in psychotropic medications for different target groups,

and generally in further psychiatric or somatic disorders as well as in legal questions. It was suggested to offer the theoretical content first and to discuss practical cases in a separate session about two weeks later.

Participants suggested that other representatives did not attend due to the costs, lack of time and heavy workload. Implementation seems to be easier without study conditions.

Coordinators from the cooperating institutions rated the contents of the training as relevant, not only for professional representatives but also for volunteers and other persons involved (e.g. judges). Some suggested making the training mandatory in order to become a legal representative. Especially the knowledge on decision-making concepts was rated as important. An implementation seems possible but it was suggested to offer a shorter version (only one day).

Quality criteria

The PRODECIDE education program is evidence based. Content and learning materials are updated regularly according to the current evidence. Legal and political developments (e.g. definition of qualification requirements for legal representatives) are taken into account.

The practical orientation is ensured. The target group was involved in the development process. The contents present relevant healthcare decisions. Moreover, the knowledge on decision-making concepts (module A) can be applied to a wide range of decisions in legal representation.

The education program is standardized: timetable (2 or 3 days), moderation cards, learning materials and presentations. A train-the-trainer course could be offered. Required qualifications of potential trainers would be defined.

The training offer is transparent regarding goals, contents, timetable, costs and qualification of trainers.

Participants receive a certificate after attending the education program, including a description of contents, the date and duration.

To ensure the quality of the program, development and evaluation followed scientific standards. Information is available on the website www.prodecide.de.

These standards will be applied in further developments. Due to the modular structure, it seems feasible to include further themes or healthcare decisions, to transfer the program into a blended or e-learning format, and to integrate it into other qualification programs for legal representatives.

Concept for e-learning modules

We outlined different ideas for the transfer of the PRODECIDE education program into a blended or e-learning format. In addition to in-class trainings, an online database with further information materials, especially on psychotropic medication, could be offered. Because of the modular structure of the program, a blended learning format seems feasible. For participants without experience, the first module, including an introduction into the e-learning system, could be offered in-class and the following modules online. In this concept, it would be possible to expand the program. Participants could then choose between themes or attend over a longer period to work on all the modules. Another option is to present all learning contents online, but offer personal meetings to discuss case examples.

For the implementation of e-learning modules, we would use the OpenOlat [32] or a comparable learning management system, which comprises specific tasks to realize complex learning and teaching scenarios (e.g., tools to create and edit content and to communicate).

As an example, we transferred the module on PEG into the OpenOlat system of the University of Hamburg. The module is structured according to the four phases of the model of Salomon [31]. The aims of the first phase are to safeguard the access to the learning content, to gain

first experiences with the navigation and to arouse motivation. The task included navigating to the forum and a first contact to other participants. In phase 2, a further interaction between participants is intended and the first content on PEG is provided. A multiple-choice test is integrated to assess the previous knowledge of participants, aiming to give them an orientation about which contents they should elaborate in depth. General information on a PEG are provided in a short video. In phase 3, participants access, structure and exchange information. Therefore, different tools are integrated, such as videos, conceptual maps and notebooks. It is a combination of self-learning and collaborative learning. In phase 4, participants work on case examples. The aim is to apply the knowledge on decision-making concepts and relevant information to make an informed decision together with or on behalf of a person with dementia.

Five persons (3 female, between 31 and 50 years old) took part in the usability tests. Four participants had already experience with e-learning. The level of education was high (>10 years school education n=4; university degree n=2). It was an iterative process of testing, analysing, revising and further testing. For analysis, we used the usability criteria described by Nassar [35]. The results regarding the seven categories are described in the following.

Consistency

Participants mentioned different colors and types of writing in the sections - it was “surprising”. They missed uniform script form, large enough to read easily. Buttons such as “closing” or “cancel” were not in the expected or usual position. Participants perceived operations as being not intuitive. Regarding the menu navigation, participants asked for more structure and a better overview, e.g. supported by pictures. The current position, sequences and procedures were not clear enough. The tasks were integrated in the menu. Participants

were uncertain whether and when to process with the tasks. It was irritating that there was a task right at the beginning without prior information.

User control

Backward and forward navigation was not intuitive. Participants were not sure how to get back to the starting page or how to proceed after single tasks. Clearly arranged pages are important and all the relevant information should be visible without scrolling or forward-backward navigation. For the navigation between pages, participants asked for more hints and clearly visible links in the text. The navigation bar was helpful.

Ease of learning

The system was not familiar to the participants. At the beginning it was not easy for them to remember how to use it but it got easier after a few attempts.

Flexibility

The system was not rated as being very flexible or adaptive. Participants perceived the different pathways offered as irritating and not helpful.

Error management

Error management was recognized as being positive, e.g. hints on saving contents or within the multiple-choice test (MC test).

Reduction of excess

Participants made suggestions for shortening the text. Especially on the first page, there was too much information, which was perceived as unnecessary. Explanations should be short or supported by videos. Doublings and functions not needed for the tasks (e.g. attach file) should be deleted.

Visibility of system status

Information on progress was rated as helpful. It should always be clear who has access to the data provided by the participants.

In conclusion, the results provide important aspects that will be taken into consideration in the further development and implementation of an e-learning concept. One problem was the integration of external formats, which contributed to user problems both in terms of lack of consistency and confusing navigation. In future, it should be examined whether the integration is necessary, how it can be done in the best possible way or whether there are alternatives within the learning platform. The module was divided into individual, building-up phases. However, the participants had the opportunity to navigate flexibly between the phases. This should allow for the different requirements and backgrounds. However, even with experience in e-learning, participants lost track and asked for better guidance. Therefore, a more stringent menu navigation and structured learning sequences should be applied.
